# Speech features as potential objective assessment indicators in schizophrenia patients with violence-related symptoms

**DOI:** 10.3389/fpsyt.2026.1906742

**Published:** 2026-09-03

**Authors:** Junjie Wang, Xindi Ling, Shujian Wang, Wen Li, Qinting Zhang, Weixiong Cai, Ningning Li, Haozhe Li

**Affiliations:** 1 Seventh People’s Hospital, Affiliated Mental Health Center, Zhejiang University School of Medicine, Hangzhou, Zhejiang, China; 2 Shanghai Key Lab of Forensic Medicine, Key Lab of Forensic Science, Ministry of Justice, Shanghai Forensic Service Platform, Academy of Forensic Science, Shanghai, China; 3 Shanghai Hongkou Mental Health Center, Mental Health Center Affiliated to Shanghai University School of Medicine, Shanghai, China

**Keywords:** formant, fundamental frequency, schizophrenia, speech feature, violent behavior

## Abstract

**Introduction:**

Schizophrenia is a chronic mental disorder characterized by impairments that can significantly disrupt daily functioning, particularly in speech and language. Speech features serve as valuable indicators of mental disorders. This study aimed to identify potential speech features that reflect the clinical symptoms, particularly violence-related symptoms in patients with schizophrenia.

**Methods:**

The present study enrolled 25 schizophrenia patients with violent behaviors, 25 schizophrenia patients without violent behaviors and 25 healthy controls. Psychiatric scales were used to assess symptom severity. Participants completed a standardized reading task consisting of numbers, ancient poems, nursery rhymes, and weather forecasts. Speech features were extracted using Praat, including fundamental frequency (F0), formant frequencies (F1–F4), formant bandwidths (B1–B4) and energy.

**Results:**

Significant differences were observed in MOAS, HCR-CV, PANSS, and BPRS scores among the three groups, indicating variations in violent behavior and the severity of psychiatric symptoms. After adjusting for years of education, age, and gender, F0, F1, F2, F3, F4, B1, B3 and B4 differed significantly among the three groups. However, LSD *post-hoc* comparisons revealed no significant difference between patients with and without violent behaviors. After partial correlations analysis, F0, F2, F3, B1, B2 and B4 showed significant correlations with certain psychiatric scale scores.

**Discussion:**

These preliminary findings suggest that fundamental frequency, partial formant frequencies and bandwidths could be potential indicators in schizophrenia. Some speech features, particularly F0, could be promising markers for violence-related symptoms in schizophrenia patients.

## 1 Introduction

Schizophrenia is a chronic mental disorder characterized by disturbances in thought, perception, emotion, and behavior, which can significantly impair daily functioning and social integration (1). Dysfunctions in speech and language are commonly seen in patients with schizophrenia (2). Moreover, previous studies have shown that individuals with mental disorders may have an increased risk of violence, which has raised global concern (3). The factors contributing to violence in patients with mental disorders are likely multifaceted, encompassing genetic susceptibility, neurobiochemical and neuroendocrine dysregulations, structural and electrophysiological brain abnormalities, personality characteristics, and social support networks (4). Currently, scale assessments are the most commonly used methods for evaluating violence risk in clinical practice. However, due to the complexity of the etiology of violence in patients with mental disorders mentioned before, these scales are limited in scope, addressing only a select number of variables while leaving other potentially meaningful factors unexamined. Given that violence can threaten social stability, greater attention should be paid to the prevention of violence among patients with schizophrenia. Accordingly, there is an urgent need to develop a violence risk assessment framework that integrates biopsychosocial dimensions, along with more precise and efficient methodologies for the evaluation and prediction of violent behavior.

Speech features were commonly used as indicators of mental health, especially in the domain of cognition (5). According to a recent review, speech features can be categorized into four types: prosodic characteristics, spectral characteristics, temporal characteristics and statistical measures (6). Among these, the first, second, and third order formant frequencies (F1, F2, F3) were commonly analyzed. In a prior study, spectral features were considered to be associated with the severity of negative symptoms in patients with schizophrenia (7). F1 is a speech feature that indicates the degree of mouth opening, whereas F2 reflects the position of the tongue. F1-F3 reflect the first, second, and third peaks in the spectrum, respectively. It was hypothesized that patients with negative symptoms may have lower variability in F1 and F2 (7). A previous study indicated that F2 showed the most pronounced reduction during speech tasks, followed by F1 (8). In addition, some prosodic characteristics such as F0 was also used in the speech feature studies. F0, also known as fundamental frequency, is a speech feature that defined as the rate at which the vocal folds vibrate. It was indicated that schizophrenia patients showed reduced accuracy and sensitivity at certain fundamental frequency deviants (9). Fundamental frequency has been considered as an indicator of cognitive load in both schizophrenia patients and healthy controls (10). However, other studies have reported no significant differences in speech features between the two groups (11).

It was observed that schizophrenia patients with flat affect tend to produce less speech and exhibit reduced speech inflection (12). Previous studies have further demonstrated the relationship between acoustic variables and negative symptoms in schizophrenia patients (7, 13). Acoustic variables, particularly spectral and temporal features, have been shown to correlate with the severity of these negative symptom dimensions (14). Specifically, formant frequencies (F1, F2, F3)—which reflect vocal tract configuration—often exhibit reduced variability in patients with prominent negative symptoms, suggesting motor speech slowing or reduced articulatory precision (15). Similarly, fundamental frequency (F0), an indicator of vocal fold vibration rate and cognitive load, has been linked to emotional blunting and cognitive deficits in schizophrenia (16). Given that many negative symptoms of schizophrenia, such as lack of emotion and poor speech, are associated with specific speech characteristics, these features have been considered as potential biomarkers for the prediction and diagnosis of schizophrenia (17, 18). Furthermore, these verbal features can be used to distinguish patients with depression from patients with schizophrenia (19).

However, focusing solely on negative symptoms provides an incomplete theoretical framework for understanding violence in schizophrenia. A previous study has expanded the scope of speech analysis to include positive symptoms and broader psychopathology (20). Positive symptoms, such as unusual thought content, hostility, and conceptual disorganization, are more directly implicated in the escalation of aggressive behavior than negative symptoms (21). For example, increased speech rate variability, irregular pauses, and heightened F0 fluctuations may reflect underlying agitation, paranoid ideation, or impaired impulse control (22).

Some symptoms of schizophrenia patients may be associated with emotional states (23). A previous study has considered speech features as biomarkers for evaluating emotions (24). Among these features, fundamental frequency is commonly regarded as an indicator of emotional state and has been utilized to analyze emotional features (25). Furthermore, emotional changes may contribute to violent behavior in these patients (26). Given that emotional changes and specific symptom dimensions contribute to violent behavior, and that speech features are sensitive to both emotional states and cognitive load, we hypothesize that distinct acoustic patterns may characterize schizophrenia patients with a history of violent behavior. While prior research has predominantly examined general schizophrenia populations or focused on negative symptomatology, this study specifically targets the intersection of speech acoustics and violence risk. This study aims to identify potential speech features that reflect the symptoms, particularly violence-related symptoms in patients with schizophrenia, providing an objective basis for clinical assessment and ultimately facilitating improved risk assessment and personalized intervention strategies.

## 2 Materials and methods

### 2.1 Participants

Based on the International Statistical Classification of Diseases and Related Health Problems, Tenth Revision (ICD-10), 50 patients with a diagnosis of schizophrenia were enrolled, including 25 with violent behaviors and 25 without violent behavior history. Patients with or without violent behaviors were recruited from inpatient services. A group of healthy controls (n = 25) with comparable age and gender distribution to the schizophrenia group was also enrolled. All other demographic variables were matched across groups.

The inclusion criteria of schizophrenia patients with violent behaviors were as follows: (1) aged 18–65 years, right-handed, (2) with a diagnosis of schizophrenia according to ICD-10, (3) first-episode, drug-naïve patients or those who had been medication-free for at least four weeks, (4) had a history of violent behavior in the last week. The inclusion criteria of schizophrenia patients without violent behaviors were as follows: (1) aged 18–65 years, right-handed, (2) with a diagnosis of schizophrenia according to ICD-10, (3) first-episode, drug-naïve patients or those who had been medication-free for at least four weeks, (4) had no history of violent behavior. Exclusion criteria included: (1) presence of other mental disorders or severe physical illnesses such as epilepsy, (2) had a history of alcohol, drug, or other substance abuse, (3) inability to complete the full experimental procedure. Matched healthy controls were selected according to the following criteria: (1) aged 18–65 years, right-handed, (2) without mental disorders.

All participants voluntarily took part in the present study. The studies were approved by the Ethics Committee of Academy of Forensic Science (2024-033). The participants provided their written informed consent to participate in this study.

### 2.2 Scale assessment

Psychiatric scales were used in the present study as follows: (1) positive and negative syndrome scale (PANSS), (2) Historical, Clinical, Risk Management - Chinese Version (HCR-CV), (3) brief psychiatric rating scale (BPRS), (4) modified overt aggression scale (MOAS).(5) Psychopathy Checklist-Revised (PCL-R) – administered only to the schizophrenia group as an exploratory measure.

### 2.3 Speech recording and acoustic feature extraction

Speech samples were recorded in a quiet room using a standardized microphone positioned approximately 15–20 cm from the participant’s mouth. All recordings were obtained with the same device and saved in uncompressed WAV format. The sampling rate, bit depth, and recording channel were kept constant across participants. Before feature extraction, recordings were inspected for artifacts, background noise, and incomplete reading. Non-speech segments and obvious recording artifacts were removed using a standardized preprocessing procedure. Acoustic features were extracted using Praat (version 6.1.15). Fundamental frequency (F0) was estimated via the autocorrelation method with a pitch floor of 40 Hz and a pitch ceiling of 500 Hz. Voicing detection parameters were set as follows: voice threshold = 0.45, silence threshold = 0.03, octave cost = 0.01, octave jump cost = 0.35, voiced/unvoiced cost = 0.14, and smoothing bandwidth = 5 Hz. Formant frequencies (F1–F4) were extracted using linear predictive coding (LPC) with the maximum formant frequency set to 6000 Hz and the number of formants fixed at 4. Corresponding formant bandwidths (B1–B4) were simultaneously derived from the same LPC model under identical analysis conditions. All acoustic analyses employed a 25 ms analysis window and pre-emphasis above 50 Hz (factor = 0.98). Identical parameter settings were applied uniformly across all participants regardless of gender, as the selected ranges adequately encompassed the vocal characteristics of both male and female speakers in our sample.

### 2.4 Study procedure

Participants were enrolled according to the inclusion and exclusion criteria. Basic demographics were collected by researchers. The PANSS and BPRS were used to assess the severity of psychiatric symptoms, the MOAS was used to evaluate violent behavior, and the HCR-CV was used to estimate clinical violence risk. Participants were asked to read a neutral corpus, containing numbers, ancient poems, nursery rhymes and weather forecasts. Finally, the obtained speech data were analyzed and processed. Among all the speech features, we selected first to fourth formant frequencies (F1-F4), first to fourth formant bandwidths (B1-B4), energy and fundamental frequency (F0) to further analyze.

### 2.5 Statistical analysis

All data were expressed as mean ± standard deviation (SD). Chi-square test was used to compare gender distribution among the three groups. One-way ANOVA was employed to compare scale scores among the three groups.

Analysis of Covariance (ANCOVA) was conducted to compare speech features among the three groups while controlling for years of education, age, and gender. The energy, fundamental frequency (F0), first to fourth formant frequencies (F1-F4) and first to fourth formant bandwidths (B1-B4) were considered as a family of tests and the Benjamini-Hochberg method was applied to control the False Discovery Rate (FDR). Considering that Fisher’s least significant difference (LSD) test is highly sensitive and the number of groups in this study was limited to three, following a significant main effect in the ANCOVA, *post hoc* multiple comparisons were conducted using Fisher’s LSD test, with pairwise comparisons based on estimated marginal means.

Partial Spearman correlation analysis was conducted within the violent schizophrenia group to examine the relationships between speech features and scale scores (MOAS, HCR-CV subscales, and PCL-R), while controlling for years of education, age, and gender. PCL-R was additionally assessed in the violent schizophrenia group as an exploratory measure of psychopathic traits, though it was not part of the main inclusion criteria. The speech features and scale scores were considered as a family of tests and the Benjamini-Hochberg method was applied to control the False Discovery Rate (FDR).

Statistical analyses were performed using SPSS (version 26.0, IBM Corp.). The threshold for statistical significance was set at *P* < 0.05.

## 3 Results

### 3.1 Demographic characteristics

In this study, no significant differences in age and gender were observed between the three groups (F = 1.15, *P* = 0.324, *χ²* = 1.299, *P=*0.522), but years of education were significantly lower in the schizophrenia group (F = 3.40, *P* = 0.039), as shown in **Table 1**.

**Table 1:** Results of demographic characteristics and scale assessments in schizophrenia patients and controls.

Demographic and scale data	Schizophrenia patients		Controls	*F/χ^2^*	*P*
	With violent behaviors	Without violent behaviors			
Age in year	37.28 ± 8.26	36.96 ± 8.14	34.24 ± 6.93	1.15^a^	0.324
Gender					0.522
male	16	14	12	1.299^b^	
female	9	11	13		
Education in year	11.00 ± 4.37	10.72 ± 4.27	13.44 ± 3.47	3.40^a^	0.039
MOAS	16.24 ± 5.59	0.08 ± 0.28	0.00 ± 0.00	209.29*^a^*	<0.001
HCR-CV	16.24 ± 5.95	6.20 ± 4.70	0.00 ± 0.00	87.57*^a^*	<0.001
H	8.80 ± 3.46	5.32 ± 3.90	0.00 ± 0.00	54.11*^a^*	<0.001
C	5.80 ± 2.57	0.88 ± 1.20	0.00 ± 0.00	91.29*^a^*	<0.001
R	1.64 ± 1.15	0.00 ± 0.00	0.00 ± 0.00	50.81*^a^*	<0.001
PANSS	56.20 ± 14.59	37.80 ± 8.12	30.68 ± 0.99	46.50*^a^*	<0.001
P	23.36 ± 7.75	7.44 ± 0.77	7.00 ± 0.00	107.41*^a^*	<0.001
N	10.12 ± 3.42	9.96 ± 3.47	7.00 ± 0.00	10.41*^a^*	<0.001
G	22.72 ± 6.19	20.40 ± 4.76	16.68 ± 0.99	11.24*^a^*	<0.001
BPRS	25.12 ± 4.78	24.96 ± 4.90	20.76 ± 1.13	10.11*^a^*	<0.001
PCL-R	13.96 ± 8.30	4.04 ± 3.93	0.04 ± 0.20	41.22*^a^*	<0.001

^a^ One-way ANOVA.

^b^ Chi-square test.

### 3.2 Scale assessment scores

The results of MOAS, HCR-CV, PANSS and BPRS are presented in **Table 1**. The result of MOAS indicated that overt aggression was observed in schizophrenia patients with violent behavior, whereas no overt aggression was observed in schizophrenia patients without violent behaviors and healthy controls. The result of HCR-CV showed that participants with schizophrenia with a history of violent behavior still presented with clinical symptoms at the time of assessment. Furthermore, they may also be at risk for future violence. However, the non-violent schizophrenia group exhibited milder symptoms compared to the violent group. The result of PANSS showed that schizophrenia patients with violent behaviors exhibited higher scores on positive subscale, negative subscale, and general psychopathology subscale than the non-violent schizophrenia group and healthy controls. This indicated that schizophrenia patients with violent behaviors were more likely to experience both positive symptoms, such as delusions and excitement, and negative symptoms, including blunted affect, emotional withdrawal, and difficulty in abstract thinking. Additionally, general symptoms such as anxiety, tension, and motor retardation were also observed in the present study. The BPRS yielded similar findings, with the psychiatric symptoms that most clearly differentiated participants with schizophrenia from healthy controls such as poor rapport, motor retardation, and blunted affect. All these scale scores showed significant difference among the three groups.

### 3.3 Speech feature analysis

**Figure 1:**
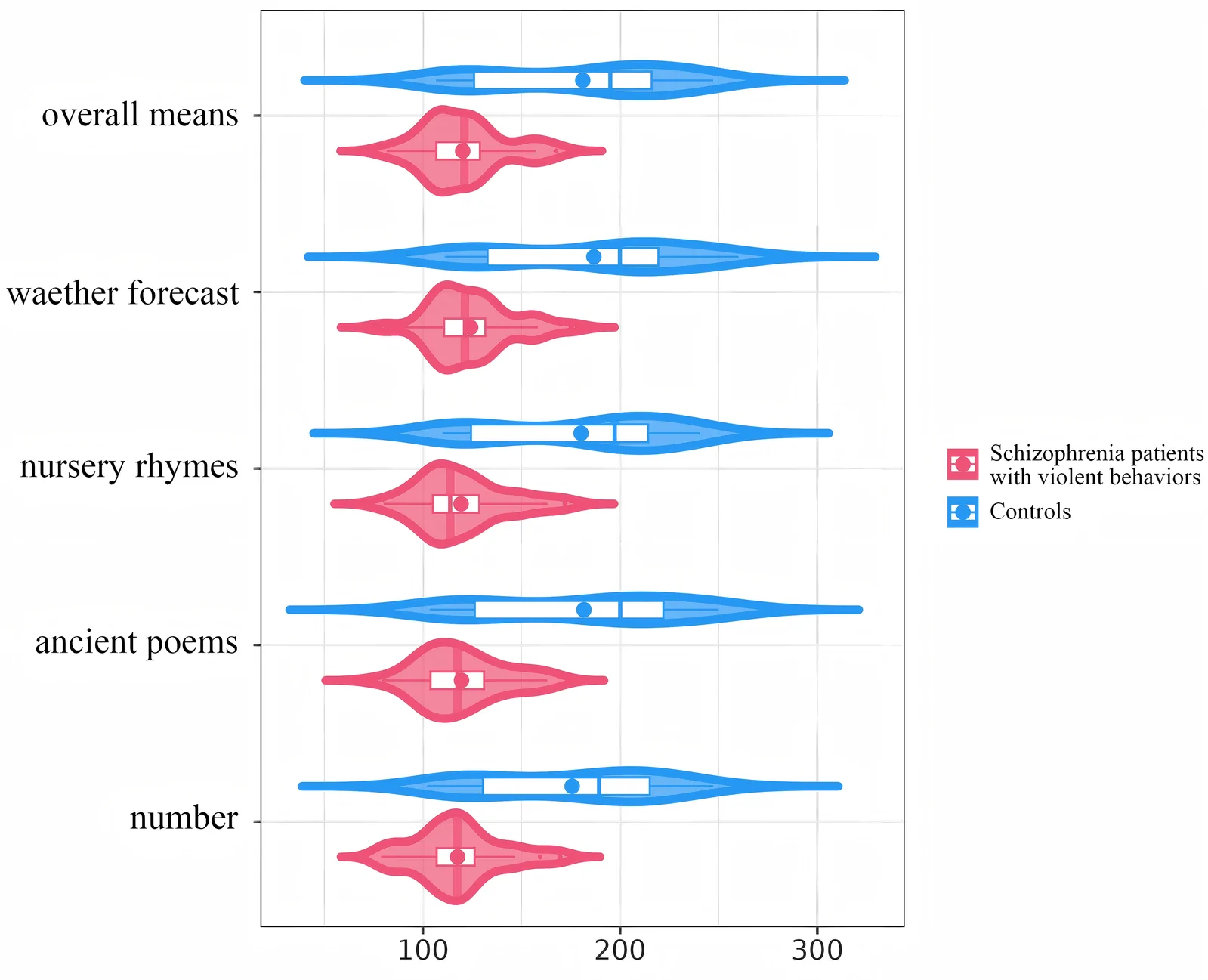
The fundamental frequency throughout the entire research process.

First, we compared speech features among the three groups across the entire speech sample. The results are presented in **Table 2**. Furthermore, we divided our analysis into four parts and analyzed differences in speech features when the participants read numbers, ancient poems, nursery rhymes and weather forecast, respectively. The results are shown in **Tables 3**–**6** and **Figure 1**.

**Table 2:** Results of speech features across the entire speech sample.

Speech features	Schizophrenia patients		Controls^c^	*P_adjust_*	η^2p^	Covariate η^2p^		
	With violent behaviors^a^	Without violent behaviors^b^				Gender	Age in year	Education in year
Energy	3.36 ± 3.11	3.1 ± 3.76	4.31 ± 8.65	0.521	0.014	0.003	0.001	<0.001
F0	122.7 ± 18.5	120.64 ± 18.42	181.06 ± 46.98	<0.001**	0.536	0.107*	0.003	0.002
F1	945.1 ± 111.97	946.91 ± 114.45	949.61 ± 97.4	<0.001**	0.077	0.045*	0.011	<0.001
F2	2517.55 ± 155.3	2524.21 ± 159.57	2424.72 ± 124.06	<0.001**	0.141	0.003	0.004	0.002
F3	3940.43 ± 116.34	3951.24 ± 120.35	3873.58 ± 107.77	<0.001**	0.192	0.011	0.009	0.045*
F4	4953.46 ± 259.87	4946.17 ± 262.2	5030.46 ± 196.56	0.004**	0.057	0.023*	<0.001	0.014*
B1	653.04 ± 97.13	656.94 ± 89.94	606.2 ± 103.7	<0.001**	0.245	0.090*	0.088*	0.013
B2	872.52 ± 99.67	878.08 ± 102.28	845.2 ± 84.94	0.121	0.029	<0.001	0.008	0.003
B3	869.55 ± 106.78	875.63 ± 115.99	879.72 ± 87.09	0.003**	0.058	0.008	0.002	0.048
B4	1092.08 ± 191.64	1119.22 ± 212.33	975.27 ± 137.85	<0.001**	0.120	0.011	0.015*	<0.001

LSD: Energy a vs b *P* = 0.953, a vs c *P* = 0.155, b vs c *P* = 0.140.

F0 a vs b *P* = 0.389, a vs c *P* < 0.001*, b vs c *P* < 0.001*.

F1 a vs b *P* = 0.666, a vs c *P* = 0.146, b vs c *P* = 0.063.

F2 a vs b *P* = 0.918, a vs c *P* < 0.001*, b vs c *P* < 0.001*.

F3 a vs b *P* = 0.578, a vs c *P* < 0.001*, b vs c *P* < 0.001*.

F4 a vs b *P* = 0.772, a vs c *P* = 0.038*, b vs c *P* = 0.071.

B1 a vs b *P* = 0.569, a vs c *P* < 0.001*, b vs c *P* < 0.001*.

B2 a vs b *P* = 0.835, a vs c *P* = 0.050, b vs c *P* = 0.077.

B3 a vs b *P* = 0.738, a vs c *P* = 0.463, b vs c *P* = 0.293.

B4 a vs b *P* = 0.259, a vs c *P* < 0.001*, b vs c *P* < 0.001*.

*P < 0.05.

**P < 0.05 after FDR correction.

**Table 3:** Results of speech features during reading numbers.

Numbers	Schizophrenia patients		Controls^c^	*P_adjust_*	η^2p^	Covariate η^2p^		
	With violent behaviors^a^	Without violent behaviors^b^				Gender	Age in year	Education in year
Energy	3.60 ± 3.19	3.15 ± 3.46	4.42 ± 8.69	0.923	0.020	0.006	0.002	<0.001
F0	121.16 ± 19.04	119.37 ± 19.07	175.68 ± 44.75	<0.001**	0.552	0.125*	<0.001	0.019
F1	949.51 ± 133.87	942.14 ± 126.05	963.13 ± 105.34	0.542	0.056	0.041	<0.001	<0.001
F2	2483.31 ± 176.99	2494.11 ± 184.59	2390.09 ± 109.69	0.067	0.136	0.008	0.002	0.003
F3	3912.70 ± 131.59	3915.97 ± 124.85	3863.58 ± 113.98	0.066	0.136	0.003	0.004	0.042
F4	4885.55 ± 347.40	4842.56 ± 335.35	5088.52 ± 167.00	0.084	0.129	0.015	0.003	0.005
B1	620.07 ± 115.27	627.20 ± 102.01	641.68 ± 136.59	0.019**	0.174	0.102*	0.072	0.015
B2	850.44 ± 101.45	857.33 ± 97.62	861.86 ± 102.58	0.827	0.030	0.006	0.021	<0.001
B3	830.92 ± 113.39	830.87 ± 124.4	940.24 ± 104.62	<0.001**	0.303	0.077*	<0.001	0.134*
B4	1094.58 ± 192.01	1113.19 ± 261.22	1007.11 ± 109.14	0.239	0.092	0.034	<0.001	0.007

LSD: Energy a vs b *P* = 0.982, a vs c *P* = 0.381, b vs c *P* = 0.370.

F0 a vs b *P* = 0.785, a vs c *P* < 0.001*, b vs c *P* < 0.001*.

F1 a vs b *P* = 0.836, a vs c *P* = 0.459, b vs c *P* = 0.586.

F2 a vs b *P* = 0.910, a vs c *P* = 0.013*, b vs c *P* = 0.010*.

F3 a vs b *P* = 0.797, a vs c *P* = 0.010*, b vs c *P* = 0.020*.

F4 a vs b *P* = 0.947, a vs c *P* = 0.022*, b vs c *P* = 0.026*.

B1 a vs b *P* = 0.772, a vs c *P* = 0.676, b vs c *P* = 0.886.

B2 a vs b *P* = 0.697, a vs c *P* = 0.908, b vs c *P* = 0.800.

B3 a vs b *P* = 0.773, a vs c *P* = 0.009*, b vs c *P* = 0.018*.

B4 a vs b *P* = 0.737, a vs c *P* = 0.136, b vs c *P* = 0.071.

*P < 0.05.

**P < 0.05 after FDR correction.

**Table 4:** Results of speech features during reading ancient poems.

Ancient poems	Schizophrenia patients		Controls^c^	*P_adjust_*	η^2p^	Covariate η^2p^		
	With violent behaviors^a^	Without violent behaviors^b^				Gender	Age in year	Education in year
Energy	2.88 ± 2.58	2.71 ± 3.27	3.66 ± 7.68	0.940	0.018	0.006	0.002	<0.001
F0	122.32 ± 18.45	120.09 ± 19.12	180.20 ± 46.12	<0.001**	0.531	0.088*	<0.001	0.003
F1	918.60 ± 121.59	922.87 ± 120.33	888.83 ± 94.41	0.014**	0.184	0.078*	0.025	0.001
F2	2532.34 ± 141.90	2538.74 ± 147.12	2450.54 ± 133.42	0.030**	0.161	0.003	0.013	0.006
F3	3932.07 ± 115.71	3955.63 ± 124.53	3878.62 ± 114.82	0.011**	0.190	0.013	0.006	0.055*
F4	4945.79 ± 251.82	4971.56 ± 233.06	4996.21 ± 222.47	0.386	0.072	0.043	<0.001	0.031
B1	634.75 ± 96.24	639.73 ± 77.92	557.64 ± 84.48	<0.001**	0.399	0.129*	0.120*	0.015
B2	822.64 ± 90.61	829.30 ± 86.55	791.96 ± 68.13	0.315	0.080	0.003	0.044	<0.001
B3	846.45 ± 105.72	859.55 ± 107.27	843.57 ± 67.66	0.322	0.080	0.002	0.016	0.034
B4	1107.98 ± 202.41	1145.09 ± 207.17	967.64 ± 162.30	0.039**	0.153	0.011	0.029	<0.001

LSD: Energy a vs b *P* = 0.950, a vs c *P* = 0.465, b vs c *P* = 0.502.

F0 a vs b *P* = 0.651, a vs c *P* < 0.001*, b vs c *P* < 0.001*.

F1 a vs b *P* = 0.724, a vs c *P* = 0.112, b vs c *P* = 0.055.

F2 a vs b *P* = 0.875, a vs c *P* = 0.009*, b vs c *P* = 0.006*.

F3 a vs b *P* = 0.415, a vs c *P* = 0.010*, b vs c *P* = 0.001*.

F4 a vs b *P* = 0.674, a vs c *P* = 0.628, b vs c *P* = 0.932.

B1 a vs b *P* = 0.897, a vs c *P* = 0.001*, b vs c *P* = 0.001*.

B2 a vs b *P* = 0.841, a vs c *P* = 0.280, b vs c *P* = 0.372.

B3 a vs b *P* = 0.762, a vs c *P* = 0.248, b vs c *P* = 0.151.

B4 a vs b *P* = 0.370, a vs c *P* = 0.095, b vs c *P* = 0.013*.

*P < 0.05.

**P < 0.05 after FDR correction.

**Table 5:** Results of speech features during reading nursery rhymes.

Nursery rhymes	Schizophrenia patients		Controls^c^	*P_adjust_*	η^2p^	Covariate η^2p^		
	With violent behaviors^a^	Without violent behaviors^b^				Gender	Age in year	Education in year
Energy	3.41 ± 3.04	3.18 ± 3.81	5.13 ± 11.54	0.900	0.023	0.004	0.002	0.001
F0	121.45 ± 18.54	119.45 ± 17.93	181.67 ± 50.13	<0.001**	0.550	0.127*	0.001	0.002
F1	945.68 ± 82.4	950.58 ± 90.32	981.31 ± 72.76	0.286	0.084	0.030	0.054	<0.001
F2	2544.81 ± 164.57	2539.37 ± 162.17	2410.41 ± 130.18	0.008**	0.199	0.001	0.006	<0.001
F3	3984.50 ± 103.19	3983.38 ± 117.65	3881.65 ± 98.67	<0.001**	0.340	0.032	0.035	0.060*
F4	5011.43 ± 179.02	5007.33 ± 215.76	5048.50 ± 175.43	0.519	0.058	0.037	0.001	0.018
B1	672.22 ± 82.07	672.20 ± 80.81	578.73 ± 84.88	<0.001**	0.405	0.118*	0.100*	0.032
B2	948.24 ± 90.13	960.17 ± 101.91	889.73 ± 72.72	0.022**	0.170	0.016	0.048	0.021
B3	907.73 ± 95.27	915.26 ± 113.88	880.66 ± 74.41	0.239	0.091	0.007	0.002	0.038
B4	1085.34 ± 182.54	1118.17 ± 191.77	971.50 ± 131.37	0.023**	0.169	<0.001	0.050	0.008

LSD: Energy a vs b *P* = 0.934, a vs c *P* = 0.435, b vs c *P* = 0.390.

F0 a vs b *P* = 0.630, a vs c *P* < 0.001*, b vs c *P* < 0.001*.

F1 a vs b *P* = 0.551, a vs c *P* = 0.478, b vs c *P* = 0.886.

F2 a vs b *P* = 0.744, a vs c *P* = 0.001*, b vs c *P* = 0.003*.

F3 a vs b *P* = 0.937, a vs c *P* < 0.001*, b vs c *P* < 0.001*.

F4 a vs b *P* = 0.957, a vs c *P* = 0.659*, b vs c *P* = 0.660*.

B1 a vs b *P* = 0.727, a vs c *P* < 0.001*, b vs c *P* < 0.001*.

B2 a vs b *P* = 0.749, a vs c *P* = 0.033*, b vs c *P* = 0.016*.

B3 a vs b *P* = 0.961, a vs c *P* = 0.068, b vs c *P* = 0.062.

B4 a vs b *P* = 0.530, a vs c *P* = 0.076, b vs c *P* = 0.019*.

*P < 0.05.

**P < 0.05 after FDR correction.

**Table 6:** Results of speech features during reading weather forecast.

Weather forecast	Schizophrenia patients		Controls^c^	*P_adjust_*	η^2p^	Covariate η^2p^		
	With violent behaviors^a^	Without violent behaviors^b^				Gender	Age in year	Education in year
Energy	3.54 ± 3.62	3.37 ± 4.50	4.03 ± 6.31	0.952	0.016	<0.001	0.006	0.006
F0	125.86 ± 18.29	123.66 ± 17.88	186.69 ± 48.96	<0.001**	0.533	0.096*	0.001	0.006
F1	966.59 ± 102.02	972.05 ± 116.62	965.17 ± 92.15	0.268	0.087	0.046	0.011	0.012
F2	2509.73 ± 132.4	2524.62 ± 142.84	2447.82 ± 118.30	0.249	0.090	0.003	0.001	0.002
F3	3932.46 ± 104.47	3950.00 ± 108.45	3870.47 ± 108.60	0.009**	0.195	0.044	0.004	0.037
F4	4971.06 ± 224.98	4963.24 ± 226.15	4988.61 ± 210.53	0.755	0.037	0.017	0.001	0.016
B1	685.11 ± 79.32	688.61 ± 86.55	646.76 ± 71.36	0.001**	0.255	0.065*	0.128*	0.003
B2	868.76 ± 70.19	865.53 ± 72.73	837.25 ± 62.77	0.207	0.097	0.023	0.038	0.006
B3	893.09 ± 96.11	896.81 ± 102.5	854.41 ± 66.19	0.333	0.078	0.005	0.001	0.028
B4	1080.42 ± 195.25	1100.44 ± 186.52	954.84 ± 145.99	0.080	0.130	0.019	0.010	<0.001

LSD: Energy a vs b *P* = 0.961, a vs c *P* = 0.722, b vs c *P* = 0.687.

F0 a vs b *P* = 0.646, a vs c *P* < 0.001*, b vs c *P* < 0.001*.

F1 a vs b *P* = 0.778, a vs c *P* = 0.392, b vs c *P* = 0.262.

F2 a vs b *P* = 0.773, a vs c *P* = 0.057, b vs c *P* = 0.030*.

F3 a vs b *P* = 0.539, a vs c *P* = 0.008*, b vs c *P* = 0.001*.

F4 a vs b *P* = 0.923, a vs c *P* = 0.695, b vs c *P* = 0.763.

B1 a vs b *P* = 0.667, a vs c *P* = 0.125, b vs c *P* = 0.053.

B2 a vs b *P* = 0.876, a vs c *P* = 0.197, b vs c *P* = 0.253.

B3 a vs b *P* = 0.963, a vs c *P* = 0.074, b vs c *P* = 0.067.

B4 a vs b *P* = 0.730, a vs c *P* = 0.053, b vs c *P* = 0.024*.

*P < 0.05.

**P < 0.05 after FDR correction.

The present study showed that, in the overall speech feature analysis, F0, F1-F4, B1, B3 and B4 differed significantly among the three groups, while energy and B2 showed no intergroup differences. After adjusting for years of education, age, and gender, the effect sizes (partial η2) of F0, F1-F4, B1, B3 and B4 were 0.536 (*P* < 0.001), 0.077 (*P* < 0.001), 0.141 (*P* < 0.001), 0.192 (*P* < 0.001), 0.057 (*P* = 0.004), 0.245 (*P* < 0.001), 0.058 (*P* = 0.003), and 0.120 (*P* < 0.001), respectively. The LSD results showed that when comparing schizophrenia patients with violent behaviors and controls, F0, F2, F3, F4, B1 and B4 differed significantly. When comparing schizophrenia patients without violent behaviors and controls, F0, F2, F3, B1 and B4 differed significantly. No other significant difference were detected among the three groups. When reading numbers, after adjusting for years of education, age, and gender, significant group differences existed in F0, B1 and B3, with the effect sizes (partial η^2^) of 0.552 (*P* < 0.001), 0.174 (*P* = 0.019) and 0.303 (*P* < 0.001), respectively. The LSD results showed that when comparing the schizophrenia patients with violent behaviors and controls, F0, F2, F3, F4 and B3 differed significantly. When comparing the schizophrenia patients without violent behaviors and controls, F0, F2, F3, F4 and B3 differed significantly. No other significant difference were detected among the three groups. During ancient poetry reading, after adjusting for years of education, age, and gender, significant differences were detected in F0, F1, F2, F3, B1 and B4, with the effect sizes (partial η^2^) of 0.531(*P* < 0.001), 0.184 (*P* = 0.014), 0.161 (*P* = 0.030), 0.190 (*P* = 0.011), 0.399 (*P* < 0.001) and 0.153 (*P* = 0.039), respectively. The LSD results showed that when comparing the schizophrenia patients with violent behaviors and controls, F0, F2, F3 and B1 differed significantly. When comparing schizophrenia patients without violent behaviors and controls, F0, F2, F3, B1and B4 differed significantly. No other significant difference were detected among the three groups. For nursery rhyme reading, after adjusting for years of education, age, and gender, F0,F2, F3, B1,B2 and B4 presented significant between-group differences, with the effect sizes (partial η2) of 0.550 (*P* < 0.001), 0.199 (*P* = 0.008), 0.340 (*P* < 0.001), 0.405 (*P* < 0.001), 0.170 (*P* = 0.022) and 0.169 (*P* = 0.023), respectively. The LSD results showed that when comparing the schizophrenia patients with violent behaviors and controls, F0, F2, F3, F4, B1 and B2 differed significantly. When comparing the schizophrenia patients without violent behaviors and controls, F0, F2, F3, F4, B1, B2 and B4 differed significantly. No other significant difference were detected among the three groups. In weather forecast reading, after adjusting for years of education, age, and gender, statistically significant differences were found in F0, F3 and B1, with the effect sizes (partial η^2^) of 0.533 (*P* < 0.001), 0.195 (*P* = 0.009) and 0.255 (*P* = 0.001), respectively. The LSD results showed that when comparing schizophrenia patients with violent behaviors and controls, F0 and F3 differed significantly. When comparing schizophrenia patients without violent behaviors and controls, F0, F2, F3 and B4 differed significantly. No other significant difference were detected among the three groups.

### 3.4 Partial correlation analysis between speech features and clinical scales

**Figure 2:**
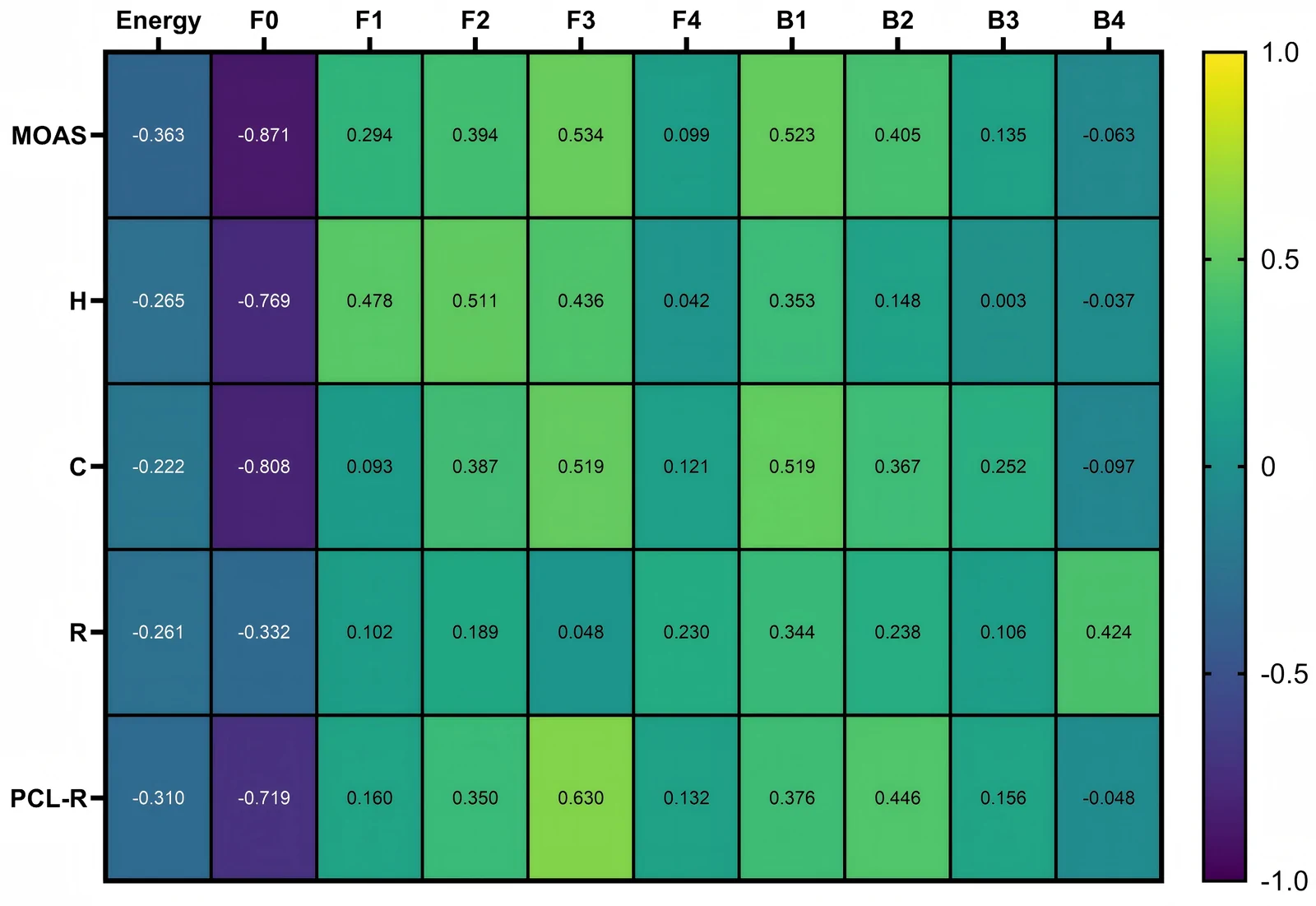
The correlation between speech features and clinical scales.

Partial correlations controlling for age and education were computed within the schizophrenia group with violent behaviors (n=25) to explore associations between key speech features (averaged across all reading tasks) and clinical scale scores. As shown in **Table 7**, several significant correlations emerged after FDR correction. Specifically, fundamental frequency (F0) showed significant negative correlations with MOAS (r = -0.871, *P* < 0.05), H (r = -0.769, *P* < 0.05), C (r = -0.808, *P* < 0.05), and PCL-R (r = -0.719, *P* < 0.05), but not with R (r = -0.332, *P* > 0.05). F1 was significantly correlated only with H (r = 0.487, *P* < 0.05). F2 was positively correlated with MOAS (r = 0.394, *P* < 0.05), H (r = 0.511, p < 0.05), C (r = 0.387, *P* < 0.05), and PCL-R (r = 0.350, *P* < 0.05). F3 showed similar positive correlations with MOAS (r = 0.534, *P* < 0.05), H (r = 0.436, *P* < 0.05), C (r = 0.519, *P* < 0.05), and PCL-R (r = 0.630, *P* < 0.05). B1 was positively correlated with all five scales: MOAS (r = 0.523), H (r = 0.353), C (r = 0.519), R (r = 0.344), and PCL-R (r = 0.376), all *P* < 0.05. Additionally, B4 showed a positive correlation only with R (r = 0.424, *P* < 0.05). B2 showed significantly positive correlation with MOAS (r=0.405, *P* < 0.05) and PCL-R (r=0.446, *P* < 0.05). No other speech features reached statistical significance after FDR correction. The results are shown as **Table 7** and **Figure 2**.

**Table 7:** Partial correlations between speech features and clinical scales in schizophrenia patients with violent behaviors.

Sccles	Energy	F0	F1	F2	F3	F4	B1	B2	B3	B4
MOAS	-0.363	-0.871**	0.294	0.394**	0.534**	0.099	0.523**	0.405**	0.135	-0.063
HCR Historical Subscale	-0.265	-0.769**	0.487**	0.511**	0.436**	0.042	0.353**	0.148	0.003	-0.037
HCR Clinical Subscale	-0.222	-0.808**	0.093	0.387**	0.519**	0.121	0.519**	0.367	0.252	-0.097
HCR Risk Management Subscale	-0.261	-0.332	0.102	0.189	0.048	0.230	0.344**	0.238	0.106	0.424**
PCL-R	-0.310	-0.719**	0.160	0.350**	0.630**	0.132	0.376**	0.446**	0.156	-0.048

Controlling for years of education, age, and gender.

**P < 0.05 after FDR correction.

HCR, Historical, Clinical, Risk Management scale.

MOAS, modified overt aggression scale.

PCL-R, Psychopathy Checklist–Revised.

## 4 Discussion

Numerous speech features have been considered valuable in the diagnosis of schizophrenia, however, previous studies have rarely focused on the schizophrenia patients with violent behaviors. Patients with mental disorders often have cognitive deficits and difficulty in perceiving emotions correctly, which may increase the likelihood of violent behaviors (27). The present study showed that the scores of the MOAS, HCR-CV and its subscales, PANSS and its subscales, and BPRS differed among the three groups, elevated current and future risk in schizophrenia patients with violent behaviors. However, scale-based assessment has certain limitations, as they may be influenced by the evaluator’s subjective judgment and are also highly labor- and time-consuming (28). Previous studies have also used biological signals such as EEG for the diagnosis of schizophrenia and other mental disorders (29). In the present study, we focused on speech features. Since the situation and conversation topic could be the factors that influence language, we chose a neutral corpus for the participants (30). Among all such features, the present study focused on fundamental frequency (F0), F1–F4, and B1–B4.

The importance of F0 has also been emphasized in previous studies. Previous meta-analysis research has indicated that the reduced F0 variability could be the minimal generalizable acoustic profile of schizophrenia (31). Additionally, a study employing both scale assessment and speech features found that F0 could serve as an objective biomarker of negative symptoms in schizophrenia patients (32). In the present study, F0 demonstrated significant discriminative value throughout the entire reading period. Regardless of whether participants were reading numbers, ancient poems, nursery rhymes, or weather forecasts, F0 showed significant differences among the three groups. After controlling for years of education, age, and gender, the three groups exhibited an extremely large main effect on F0, with the effect sizes (partial η^2^) >0.50, regardless of the corpus. This result constitutes a very large effect size, indicating that over 50% of the variance in F0 could be explained by the difference of groups. Although gender showed a significant effect, the grouping variable consistently explained more variance in F0. Previous research has demonstrated that F0 might be associated with sociolinguistic factors such as gender and age (33). However, in the present study, the result of ANCOVA suggested that F0 is highly sensitive to group identity while being relatively immune to individual background characteristics. The fundamental frequency results in the present study align with those reported in prior research. A previous machine learning study has also used F0 to detect the symptoms of schizophrenia, which achieved an accuracy of 90% (34). However, when we use LSD to compare the result of F0, significant differences were only shown between schizophrenia patients and the control group, no matter of violent symptoms. This finding may be explained by the fact that both the violent and non-violent schizophrenia patients were recruited from hospital inpatient settings, presenting with more severe symptoms than those in outpatient settings. Therefore, we suggest that F0 can be considered a promising factor for distinguishing schizophrenia patients from healthy controls.

Formants function as spectral characteristics of speech. Previous research has identified correlations between formant frequencies (F1, F2, F4, F6) and negative symptoms measured by the PANSS (35). However, those studies reported no significant differences in formant frequencies and bandwidth between the patient group and the control group. The present study achieved different results: After controlling for education, age, and gender, we found that B1 and B3 reached statistical significance when participants read numbers. Both B1 and B3, the grouping variable consistently demonstrated large effect sizes (partial η² = 0.174 and 0.303), exceeding those of gender (0.102 in B1 and 0.077 in B3) and education (0.134 in B3). Although gender and education made significant contributions, the grouping variable consistently explained more variance in B1 and B3 than the demographic covariates. During nursery rhyme reading, F2, F3, B1, B2 and B4 were significantly different, with the partial η^2^ values of 0.199, 0.340, 0.405,0.170 and 0.169, indicating a large effect size. Gender and age made significant contributions in B1 (covariate partial η² = 0.118 and 0.100), education made significant contributions in F3 (covariate partial η² = 0.060). However, these values indicate that the significance of the covariates does not override the significance of the speech features across the three groups. During ancient poems reading, we found similar results. Significant differences were found in F1, F2, F3, B1 and B4, with the partial η^2^ values of 0.184, 0.161, 0.190, 0.399 and 0.153. Although significant contributions were made by gender in F1 and B1 (covariate partial η² = 0.078 and 0.129), age in B1 (covariate partial η² = 0.120) and education in year in F3 (covariate partial η² = 0.055), we still consider that speech features explained more variance. In weather forecast reading, F3 and B1 showed significant differences (partial η^2^ = 0.195 and 0.255). Gender and age made significant contributions in B1 (covariate partial η² = 0.065 and 0.128), which cannot diminish or supersede the significance of the speech features. Previous studies investigated that F0 did not reveal any significant statistical differences, and this is suspected to be associated with education levels (36). Consequently, in the present study, we controlled for factors that might potentially affect the results by incorporating them as covariates. Findings from the current study suggest that formant frequencies and bandwidth may represent promising speech features for distinguishing schizophrenia from healthy controls. Since many individuals with mental disorders exhibit cognitive and thought impairments, these are manifested in speech disturbances (37). As a biological signal that can reveal emotions, speech features are highly correlated with functional impairment (38). Previous studies have shown that speech features can be used to assess the cognitive system, thereby differentiating individuals with mental disorders (39). The present study has also yielded similar findings. Another recent study has also suggested that spectral formants are strongly associated with negative symptoms and may be used in the assessment and intervention of schizophrenia (40). Furthermore, previous research suggested that F1 and F2 have been reported to be lower in patients who suffer more severe negative symptoms (7). Our results are only partially consistent with the findings of earlier studies. Notably, the speech parameters demonstrating significant large effects varied as a function of the reading material employed in the present study. However, irrespective of the specific reading material employed, F0 consistently produced the largest effect size in differentiating the three groups, accounting for over half of the total variance. Significant effects were also observed for F1 and F2 during the reading of certain speech materials, corroborating earlier research that has reported correlations between F0, F1, and F2 (7, 8). As for bandwidth, B1 exhibited the second largest effect size in the present study, though its value was substantially smaller than that of F0. Further research with larger samples and standardized corpora is needed to clarify the role of speech features in schizophrenia. According to our current study, speech features, especially F0, may serve as potential indicators for differentiating schizophrenia patients from healthy controls, regardless of individual background characteristics.

Within the schizophrenia group with violent behaviors, partial correlation analyses controlling for age and education revealed several significant associations. Lower fundamental frequency (F0) was consistently correlated with higher scores on MOAS, HCR-CV Historical (H) and Clinical (C) subscales, and PCL-R, indicating that reduced F0 is associated with more severe violence risk and psychopathic traits. In contrast, higher formant frequencies (F2, F3) and broader bandwidths (B1) were positively correlated with these measures. Notably, B1 correlated with all five clinical scales, including the HCR-CV Risk subscale (R), suggesting a potential link between spectral characteristics and future violence risk. Additionally, F1 was associated only with H, and B4 only with R. These exploratory findings suggest that acoustic speech features, particularly F0, F2, F3, and B1, may index clinical severity in schizophrenia patients with violent behavior. These results, to a certain degree, reveal the relationship between violence-related indicators and acoustic speech features. However, given the modest sample size and cross-sectional design, these correlations should be interpreted as hypothesis-generating rather than confirmatory.

Several potential limitations of the present study should be acknowledged. First, the number of participants was relatively small. Future studies should include larger samples to minimize the impact of individual differences. Second, only four types of speech corpora were collected, more continuous speech samples should be collected to further clarify how speech feature changes vary across different corpora. Third, schizophrenia patients with or without violent behaviors were all enrolled from hospital inpatient settings. This may have obscured the differences between the two schizophrenia groups, resulting in less prominent group disparities in the observed outcomes. Also, medication exposure, illness duration, and smoking status were not fully controlled in the present study, and these factors may influence acoustic speech production. Future studies should collect detailed clinical and pharmacological information and incorporate these variables into adjusted models.

## 5 Conclusion

The findings indicate that fundamental frequency, partial formant frequencies and bandwidths differ significantly between schizophrenia patients and healthy controls, suggesting that these speech features could be potential indicators in schizophrenia. Furthermore, specific speech features, particularly F0, emerged as potential markers for violence-related symptoms in schizophrenia. However, these results should be interpreted with caution, as they constitute preliminary evidence rather than validated predictors of violence risk, and thus warrant further validation in larger, longitudinal studies.

## Data Availability

The raw data supporting the conclusions of this article will be made available by the authors, without undue reservation.
